# Supplementary material for: A fossil assemblage from the mid–late Maastrichtian of Gavdos Island, Greece, provides insights into the pre-extinction pelagic ichthyofaunas of the Tethys
Source: PLoS One. 2022 Apr 13;17(4):e0265780. doi: 10.1371/journal.pone.0265780 (PMC9007369; doi:10.1371/journal.pone.0265780)
Supplement: Attachment — Submitted filename: Response to reviewers.docx [file pone.0265780.s002.docx]

We are indebted to the reviewers and editor for their careful and positive evaluation of our paper. Below we expand on the main comments made by reviewer 1 in their review letter and on the marked pdf, as well as on the comments made by the editorial stuff of the journal.

**Reviewer 1:**

**General comment on †Ichthyodectoidei:** We appreciated and accepted the reviewer’s suggestion to narrow down the attribution of our †Ichthyodectiform indet. We have added the reference of †Ichthyodectoidei and corrected/updated all relevant passages in the text and table 1.

**Abstract and main text**: We chose to put fishes in quotation marks, in order to make it clear that we refer to them as a paraphyletic assemblage, which includes chondrichthyans and actinopterygians but excludes sarcopterygians. We can remove the quotation marks if the editor wishes us to do so.

**Comparative material section:** As requested by the reviewer, we listed the naming authorities of various taxa and provided references for them.

In addition to the above, we accepted all writing suggestions made by the reviewer in their annotated pdf.

**Additional changes:**

**Fig 1.** We changed the color of the Pindos Unit in Fig 1B to grey, do it is not confused with the Cretaceous-aged platy limestone on Fig1C. The caption was updated accordingly.

**Fig4B.** We added a label for the dermopterotic ossification in both figure and caption
